# Improving the quality of Napier grass silage with pyroligneous acid: Fermentation, aerobic stability, and microbial communities

**DOI:** 10.3389/fmicb.2022.1034198

**Published:** 2022-11-14

**Authors:** Dandan Chen, Mingyang Zheng, Yuxin Zhou, Lin Gao, Wei Zhou, Mingya Wang, Yongwen Zhu, Weijie Xu

**Affiliations:** ^1^Guangdong Key Laboratory for Innovative Development and Utilization of Forest Plant Germplasm, State Key Laboratory for Conservation and Utilization of Subtropical Agro-Bioresources, Guangdong Province Research Center of Woody Forage Engineering Technology, Guangdong Research and Development Centre of Modern Agriculture (Woody Forage) Industrial Technology, College of Forestry and Landscape Architecture, South China Agricultural University, Guangzhou, China; ^2^College of Animal Science and Technology, Hebei Agricultural University, Baoding, China; ^3^College of Animal Science, South China Agricultural University, Guangzhou, China; ^4^Zhengzhi Poultry Industry Co., Ltd., Shantou, China

**Keywords:** pyroligneous acid, fermentation quality, microbial communities, aerobic stability, Napier grass

## Abstract

The presence of undesirable microorganisms in silage always leads to poor fermentation quality and low aerobic stability. Pyroligneous acid (PA), a by-product of biochar production, is known to have strong antimicrobial and antioxidant activities. To investigate the effects of PA on fermentation characteristics, aerobic stability, and microbial communities, Napier grass was ensiled with or without 1 and 2% PA for 30 days and then aerobically stored for 5 days. The results showed that PA application decreased (*P* < 0.01) the pH value, ammonia nitrogen content, and number of undesirable microorganisms (coliform bacteria, yeasts, and molds) after 30 days of ensiling and 5 days of exposure to air. The temperature of the PA-treated group was stable during the 5-day aerobic test, which did not exceed room temperature more than 2°C. The addition of PA also enhanced the relative abundance of *Lactobacillus* and reduced that of *Klebsiella* and *Kosakonia*. The relative abundance of *Candida* was higher in PA-treated silage than in untreated silage. The addition of PA decreased the relative abundance of *Kodamaea* and increased that of *Monascus* after 5 days of exposure to air. The abundances of *Cladosporium* and *Neurospora* were relatively high in 2% PA-treated NG, while these genera were note observed in the control group. These results suggested that the addition of PA could improve fermentation characteristics and aerobic stability, and alter microbial communities of silage.

## Introduction

Ensiling has become a universal method for preserving fresh forage and supplying moist feedstock all year round (Wang et al., 2021). With the increasing demand for livestock products, more attention has been paid to silage production, especially in developing countries. Napier grass (*Pennisetum purpureum* Schum) is an important source for manufacturing biofuel and animal feed. It is widely cultivated for ruminant feed in tropical and subtropical regions because of its short growth cycle, high biomass, and strong adaptability (Tao et al., 2021). In these regions, the main constraint restricting long-term feed supply is humidity and rain. Ensiling might be a better choice for preservation of forage to constantly provide highly palatable and nutritious feed for livestock. In general, fermentation of silage mainly depends on lactic acid bacteria (LAB), which produce organic acids (mainly lactic acid and acetic acid), for creating an acidic environment and suppress the microbial metabolism and reproduction, through which the original quality of fresh forage is preserved as much as possible (He et al., 2020a). However, the inadequate epiphytic LAB count and the low-water soluble carbohydrate (WSC) content of raw material would suppress the success of ensiling under natural fermentation conditions, thus failing to meet the daily nutritional demand of livestock (Wu et al., 2020). Consequently, *Clostridia* and *Enterobacter* would grow vigorously, causing abundant proteolysis and butyric acid accumulation. After exposure to air, aerobic spoilage of silage is also frequently observed. Yeast and *Acetobacter* would compete with LAB for substance, causing dry matter loss and spoilage (Kung et al., 2021). Undesirable microorganisms might also produce numerous secondary metabolites, including mycotoxins, which would affect the health and production of animals (Zong et al., 2022). Silage with potential safety hazards would also incur huge economic losses to farmers. Therefore, it is necessary to take some measures for improving fermentation quality and aerobic stability of silage.

Pyroligneous acid (PA), also called wood vinegar, is a brown liquid by-product of pyrolysis of biomass, which is transformed into biofuel and biochar (Zhang et al., 2019a). PA is a complex mixture of compounds and contains over 200 kinds of natural organic compounds, including organic acids, phenols, aldehydes, alcohols, esters, ketones, furan and pyran derivatives, hydrocarbons, and nitrogen compounds (Zhu et al., 2021). Due to the presence of these chemical ingredients, PA is considered organic wastewater and would impose an extreme burden on ecological environment stability (Cezary, 2015). Therefore, the effective application of PA is one of the methods of waste recycling and achieving green and sustainable development. Organic acids and phenolic compounds are the dominant compounds of PA, which have exhibited remarkable antimicrobial property. In general, organic acids account for around 30–70% of total organic compounds present in PA, 90% of which is acetic acid (Fan et al., 2022). PA with a high concentration of organic acids might inhibit the fermentation of microorganisms and reduce the nutrient loss of silage. Simultaneously, previous studies indicated that acetic acid can also improve the aerobic stability of silage. However, there are few studies on the effect of PA on fermentation quality and aerobic stability of silage.

Therefore, we hypothesized that the addition of PA could improve the fermentation quality and aerobic stability of Napier grass by inhibiting undesirable microorganisms. In the present study, Napier grass was harvested randomly and ensiled with or without 1 or 2% PA for 30-day fermentation and then aerobically stored for 5 days. Following that, the fermentation characteristics and aerobic stability were analyzed. Moreover, the change of microbial community is determined to help us comprehend the effect of PA on silage fermentation.

## Materials and methods

### Silage preparation

Dwarf Napier grass was collected from the experimental plot of South China Agricultural University, which was cultivated at a row width of 60 cm and an intrarow spacing of 40 cm for 90 days. Dwarf Napier grass was harvested in May 2021, which was immediately chopped to a length of around 2 cm using a grass cutter. After sufficient mixing, chemical compositions and microbial populations of the fresh material (FM) were determined, which were performed in triplicate. Different concentrations of PA were applied to the fresh Napier grass (approximately 200 g) and then assigned to one of the following treatments: (1) no additive (CK), (2) 1% pyroligneous acid (fresh matter basis, 1% PA), and (3) 2% pyroligneous acid (fresh matter basis, 2% PA). A total of 42 samples (3 treatments × 2 days × 7 replicates) were packed in polyethylene bags (20 × 30 cm; Dongguan Bojia Packaging; China). Subsequently, the samples were sealed in bags using a vacuum sealing machine (Lvye DZ280; Dongguan Yijian Packaging Machinery, Dongguan, China) to reach anaerobic conditions and were placed in a room with ambient temperature (25–30°C), After 30 days of ensiling, the bags were opened, and fermentation quality and microbial communities were measured on day 0 and day 5 of aerobic exposure.

### Assessing aerobic stability

The determination methods were similar to those used in our earlier study (He et al., 2020b). After 30 days of fermentation, seven bags of each treatment groups were opened, thoroughly mixed, and separated into three repetitions. Following that, 400 g of silage from each treatment groups was placed loosely into 1,000-mL plastic buckets to estimate aerobic stability, with nine buckets in total. A layer of cheesecloth was covered around the barrel to reduce moisture volatilization and potential contamination but to permit air penetration. In addition, all the barrels were placed in cartons. A layer of polystyrene foam was laid between the barrels and cartons to prevent rapid thermal loss. A thermograph was used to measure room temperature and the temperature of silage at 10-min intervals during 5 days of aerobic exposure (SMOWO MDL-1048A, Shanghai Tianhe Automation Instrument Co., Ltd. (Shanghai, China). In general, if the silage temperature exceeds the room temperature above two degrees, it is considered that the silage had underwent aerobic deterioration.

### Determination of fermentation characteristics and chemical compositions

According to Wang et al. (2021), fermentation characteristics and chemical compositions of the silage sample were determined on days 0 and 5 of aerobic exposure, respectively. A measure of 20 g of the sample was added to with 180 mL of normal sterile saline and mixed with shaking. The supernatant was gradient-diluted from 10^−1^ to 10^−6^. Serial dilutions of 1 mL were, respectively, inoculated in Man, Rogosa, and Sharpe (MRS) agar and Violet Red Bile agar to culture lactic acid bacteria (LAB) and coliform bacteria under a temperature of 30°C for 2 days (Chen et al., 2021). Meanwhile, 100 μL of the diluent was added to Rose Bengal agar and cultured for 3 days under 28°C to obtain yeasts and molds (Guo et al., 2021). Another 20 g of the sample was mixed with 180 mL distilled water and stored overnight at 4°C. Subsequently, it was filtered, pH value was measured, and organic acids and ammonia nitrogen (NH_3_-N) were analyzed. pH was determined using a glass electrode pH meter (PHS-3C, INESA Scientific Instrument, Shanghai, China). The NH_3_-N content was analyzed by using the phenol-hypochlorite colorimetric method (Broderick and Kang, 1980). Organic acids (mainly lactic acid, acetic acid, propionic acid, and butyric acid) were determined by high-performance liquid chromatography (HPLC) (column, Shodex RSpak KC-811S-DVB gel C (8.0 mm 930 cm; Shimadzu, Tokyo, Japan) under the following conditions: oven temperature: 50°C, mobile phase: 3 mmol/L HCLO_4_, flow rate: 1.0 mL/min, injection volume: 5 μL, and detector: SPD-M10AVP) (Bai et al., 2020). The remaining silage sample was dried at 65°C for 2 days to measure the content of dry matter and protein components [true protein (TP) and crude protein (CP)] (Ke et al., 2017). The CP content was measured using an automatic Kjeldahl apparatus (Kjeltec 2300 Auto Analyzer, FOSS Analytical AB, Hoganas, Sweden) according to the method of the Association of Official Analytical Chemists. At the same time, the contents of neutral detergent fiber (NDF), detergent fiber (ADF), and WSC in the fresh material samples were also determined as mentioned by Wang et al. (2019). The contents of NDF and ADF were measured using an A220 Fiber Analyzer (ANKOM Technology Corp., Macedon, NY, USA), while WSC concentration was analyzed by 3,5-dinitrosalicylic acid colorimetry.

### Microbial diversity analysis

A DNA kit (Omega Biotek, Norcross, GA, U.S.) was used for total DNA extraction in accordance with the manufacturer's instructions. The primers of 341F (CCTACGGGNGGCWGCAG) and 806R (GGACTACHVGGGTATCTAAT) were used to amplify the V3-V4 region of 16S rDNA. For fungi, the ITS region was targeted using primers ITS3_KYO2F (GATGAAGAACGYAGYRAA) and ITS4R (TCCTCCGCTTATTGATATGC). The purified polymerase chain reaction (PCR) products were sequenced using the Illumina HiSeq 2500 system. In addition, the analysis of raw sequences was performed as described in Wang et al. (2019). Finally, microbial communities were analyzed by the free online platform (http://www.omicshare.com/tools), which included alpha diversity, β-diversity, and relative abundance.

### Statistical analysis

In the present study, IBM SPSS20.0 software was used to evaluate the effects of PA and exposure time on fermentation characteristics with two-way analysis of variance (ANOVA). Duncan's test was used to compare the degree of difference between different treatments. If the *P*-value was lower than 0.05, it would be inferred to have a significant effect. The relevant figures of microbial communities were obtained by using the Omicsmart online platform, and the aerobic stability assessment diagrams were constructed by GraphPad prism 8 software. Furthermore, all of them were enhanced by Adobe Illustrator CS 6.0 software.

## Results

### Characteristics of the fresh Napier grass

The chemical compositions and microbial populations of Napier grass before silage are summarized in Table 1. The DM content was 210 g/kg FM, and the contents of CP, NDF, ADF, and WSC were 139 g/kg DM, 350 g/kg DM, 122 g/kg DM, and 57.1 g/kg DM, respectively. For microorganisms, the LAB count was 3.92 log_10_ CFU/g FM. Coliform bacteria and yeast counts were 5.15 log_10_ CFU/g FM and 3.78 log_10_ CFU/g FM, respectively. The count of molds was less than 2.00 log_10_ CFU/g FM.

**Table 1:** Characteristics of the fresh Napier grass before ensiling (±SD, *n* = 3).

**Item**	**Means ±SD**
Dry Matter (g/kg)	210 ± 10.21
Crude protein (g/kg DM)	139 ± 1.93
Neutral detergent fiber (g/kg DM)	350 ± 17.2
Acid detergent fiber (g/kg DM)	122 ± 6.83
Water-soluble carbohydrates (g/kg DM)	57.1 ± 2.47
Lactic acid bacteria (log_10_ CFU/g FM)	3.92 ± 0.14
Coliform bacteria (log_10_ CFU/g FM)	5.15 ± 0.26
Yeast (log_10_ CFU/g FM)	3.78 ± 0.16
Molds (log_10_ CFU/g FM)	<2.00

FM, fresh matter; DM, dry matter; CFU, colony-forming units; SD, standard deviation.

### Aerobic stability of Napier grass silage

**Figure 1:**
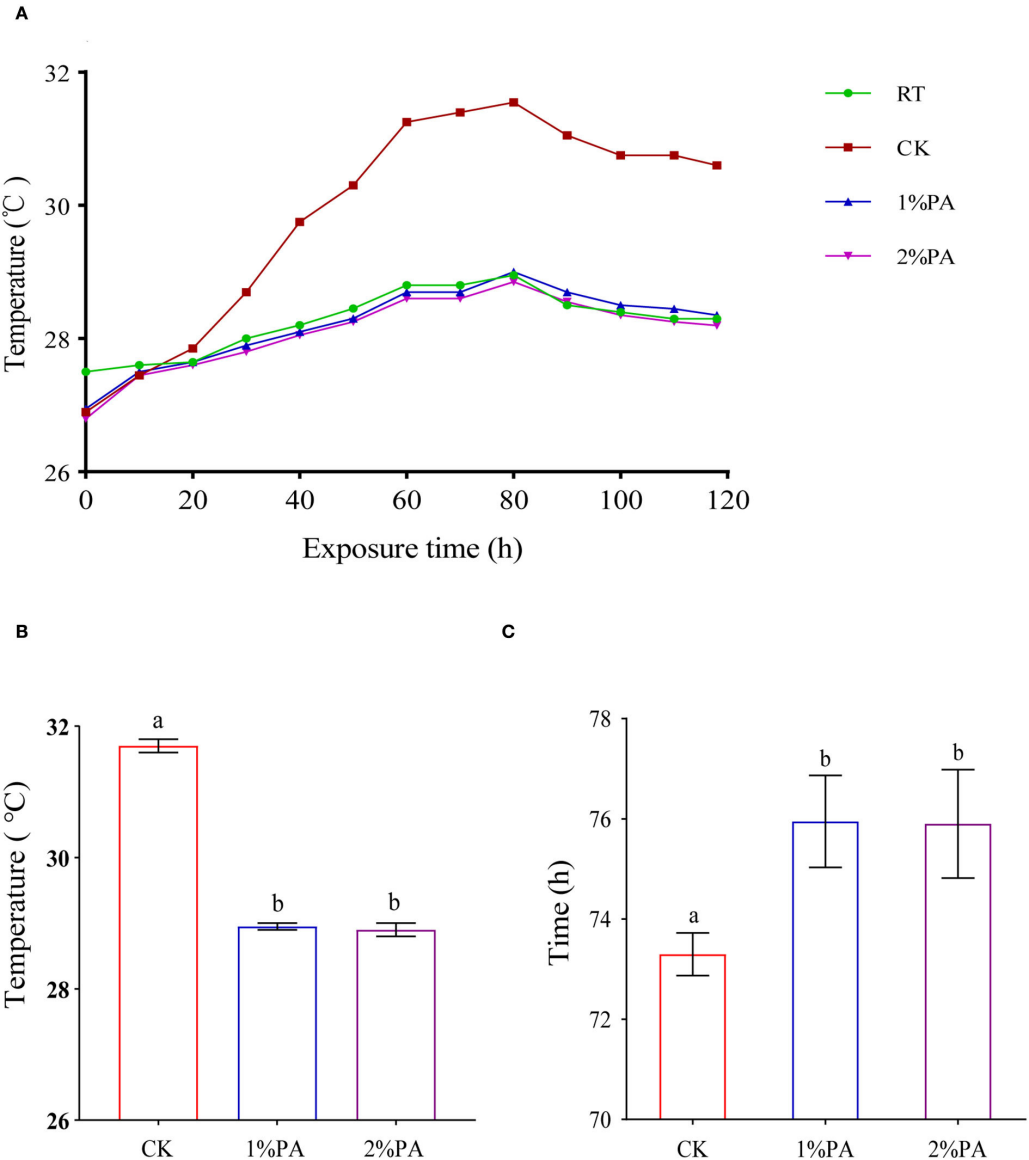
**(A)** Aerobic stability of Napier grass silages with or without 1% and 2% pyroligneous acid during the 5-day aerobic test. **(B)** Maximum temperature attained within 5 days of aerobic exposure and **(C)** the time required.

Aerobic stability, the maximum temperature attained within 5 days of aerobic exposure, and the time required are listed in Figure 1. The untreated Napier grass deteriorated after aerobic exposure of 58 h. PA-treated Napier grass showed higher aerobic stability than the untreated silage. The temperatures in PA treatments did not exceed the room temperature more than 2°C during the 5-day aerobic test and were lower than those of the control group (*P* < 0.05), which were below 29°C.

### Fermentation quality of Napier grass silage

Fermentation characteristics are shown in Table 2. PA markedly reduced the pH value (*P* < 0.01) when compared with the control group. On day 5 compared with day 0, the pH value of silage increased from 4.52 to 6.97 in the untreated silage, was unchanged in 1% PA-treated silage, and even prominently decreased in 2% PA-treated silage (*P* < 0.01). The addition of PA resulted in the increase in lactic acid and acetic acid contents (*P* > 0.01) on days 0 and 5, respectively. The numbers of yeasts and molds in all treatments were less than the detectable levels on day 0. The number of coliform bacteria was decreased by PA (*P* < 0.01), while it was relatively high in the control group. On day 5 of aerobic exposure, the numbers of yeasts and molds were significantly reduced in PA-treated silage, compared with the control group. Meanwhile the LAB count was significantly increased in the 2% PA-treated group (*P* < 0.05). The addition of PA reduced the NH_3_-N proportion (*P* < 0.01), when compared with the control group. The TP content increased from day 0 to day 5 in the untreated silage. In addition, on day 5, the TP content was higher in the control group than that in PA-treated silage.

**Table 2:** Chemical compositions and fermentation characteristics of Napier grass with or without PA treatment under aerobic exposure.

**Items**	**Days**	**Treatment**			**SEM**	***P*** **-value**		
		**CK**	**1%PA**	**2%PA**		**D**	**T**	**D × T**
Dry Matter (g/kg FM)	D0	194	193	191	0.181	0.60	0.72	0.55
	D5	191	199	195				
pH	D0	4.52^aB^	4.03^b^	4.06^bA^	0.077	<0.01	<0.01	<0.01
	D5	6.97^aA^	4.00^b^	3.83^bB^				
Lactic acid (g/kg DM)	D0	0.26	0.45	0.93	0.16	0.45	0.14	0.78
	D5	0.20	0.93	1.27				
Acetic acid (g/kg DM)	D0	0.06	0.09	0.21	0.07	0.037	0.55	0.847
	D5	0.30	0.52	0.54				
Lactic acid bacteria (log_10_ CFU/g FM)	D0	8.19	7.90	8.09^B^	0.12	0.02	0.34	0.84
	D5	9.07	8.43	8.76^A^				
Coliform bacteria (log_10_ CFU/g FM)	D0	4.15^aB^	<2.00^bB^	<2.00^bB^	0.502	0.55	<0.01	0.07
	D5	8.58^aA^	5.52^bA^	6.00^bA^				
Yeast (log_10_ CFU/g FM)	D0	<2.00^B^	<2.00^B^	<2.00^B^	0.41	<0.01	-	-
	D5	7.43^aA^	4.56^cA^	5.41^abA^				
Molds (log_10_ CFU/g FM)	D0	<2.00^B^	<2.00	<2.00	-	-	-	-
	D5	6.06^aA^	<2.00^b^	<2.00^b^				
Crude protein (%DM)	D0	11.3	11.7	11.9	0.13	0.18	0.04	0.54
	D5	10.6	11.3	11.9				
True protein (%DM)	D0	6.35^B^	6.27	6.79	0.12	0.34	0.02	0.01
	D5	7.88^aA^	5.84^b^	6.44^b^				
Ammonia nitrogen (%DM)	D0	0.121^a^	0.047^b^	0.033^c^	0.01	0.04	<0.01	0.06
	D5	0.230^a^	0.055^b^	0.041^b^				

Means in the same column (A–B) or row (a–c) followed by different letters differ (*P* < 0.05). SEM, standard error of means; DM, dry matter; CFU, colony-forming units; FM, fresh matter; D, aerobic exposure days effect; T, treatments effect; D×T, the interaction effect of treatments and aerobic exposure days; D0, aerobic exposure of day 0; D5, aerobic exposure of day.

### Microbial diversity of Napier grass silage

**Figure 2:**
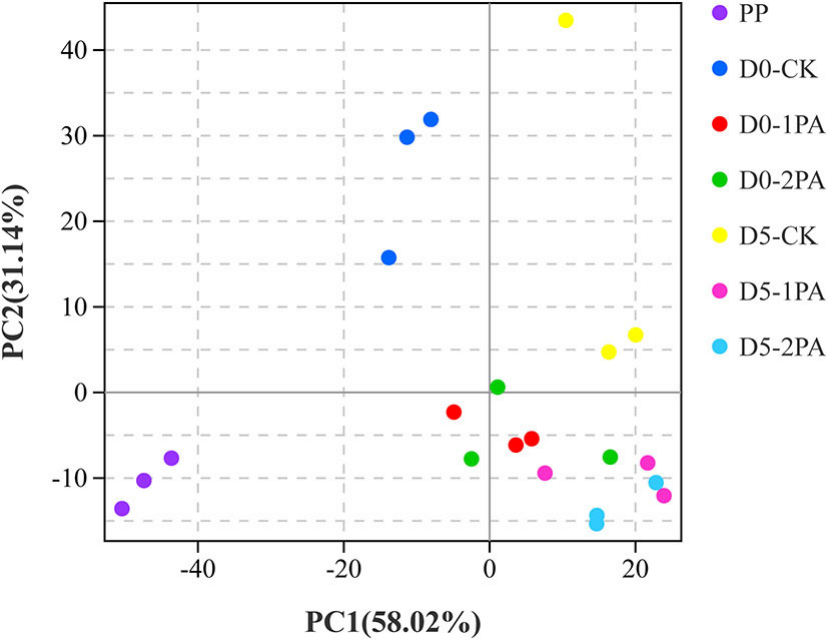
Principal component analysis of bacterial communities for Napier grass silages treated without or with 1 and 2% pyroligneous acid after 30 days of ensiling and 5 days of exposure to air.

**Figure 3:**
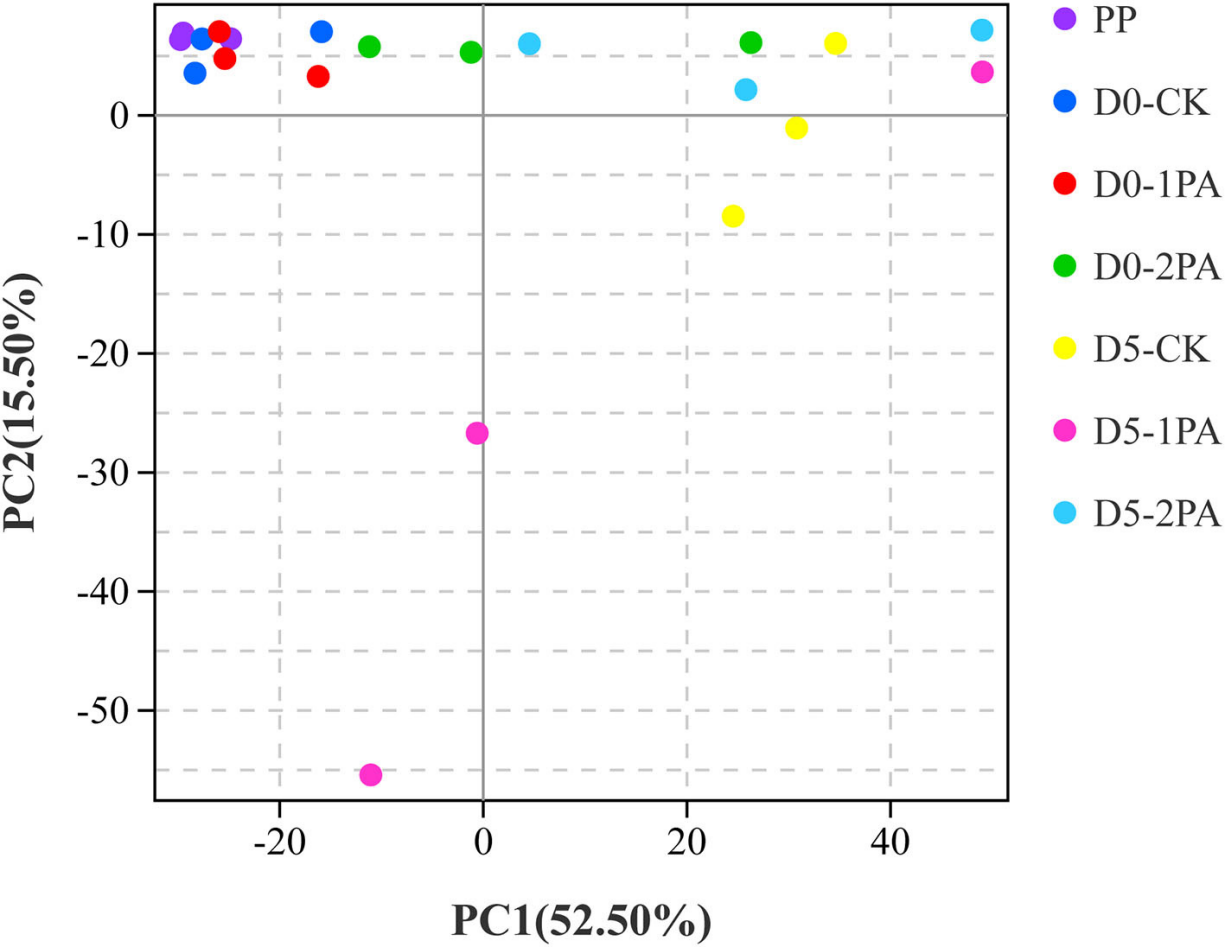
Principal component analysis of fungal communities for Napier grass silages treated without or with 1 and 2% pyroligneous acid after 30 days of ensiling and 5 days of exposure to air.

Alpha diversity of microbial communities is shown in Table 3. Good's coverage values of all treatments were greater than 0.99. For bacterial communities, Sobs, Chao1, and Ace indices were higher in PA-treated silage than in the untreated silage, while the Simpson index was opposite. The different treatments resulted in the variation of fungal communities. The addition of PA led to the decrease in Chao1, Ace, and Simpson indices, compared with the control group. The Sobs index was also decreased by 2% PA treatment. Moreover, all indices of microbial communities reduced after 5 days of exposure to air. The β-diversity of microbial communities is shown in Figures 2, 3, respectively. For bacterial communities, distinct segregation was observed between PA-treated and untreated silage, as well as in the control group on different days of aerobic exposure. However, the PA-treated samples only had a little shift in the bacterial community. For fungal communities, 1% PA-treated samples were slightly separated from the untreated samples after 30 days of ensiling. However, 2% PA-treated and untreated silage were separated from each other. After 5 days of exposure to air, clear segregation was observed among all treatment groups.

**Table 3:** Alpha diversity of microbial communities of Napier grass silage during aerobic exposure.

**α diversity**	**Item**	**Treatment**	**Sobs**	**Chao1**	**Ace**	**Simpson**	**Coverage**
Bacteria	D0	CK	208.66	230.32	224.98	0.82	1.00
		1%PA	217.00	252.57	261.22	0.74	0.99
		2%PA	220.33	257.96	261.90	0.74	1.00
	D5	CK	165.00	198.12	205.93	0.80	0.99
		1%PA	180.67	210.20	221.16	0.66	0.99
		2%PA	192.33	229.29	241.73	0.63	1.00
Fungi	D0	CK	495.67	576.33	570.65	0.93	1.00
		1%PA	482.00	544.73	538.04	0.94	1.00
		2%PA	382.33	447.77	449.99	0.79	0.99
	D5	CK	266.33	331.73	348.81	0.57	1.00
		1%PA	273.00	324.68	336.70	0.56	0.99
		2%PA	251.33	312.07	311.92	0.56	1.00

CK, control; D0, aerobic exposure of day 0; D5, aerobic exposure of day 5; 1% PA, 1% FM pyroligneous acid added; 2% PA, 2% FM pyroligneous acid added.

### Microbial abundance of Napier grass silage

**Figure 4:**
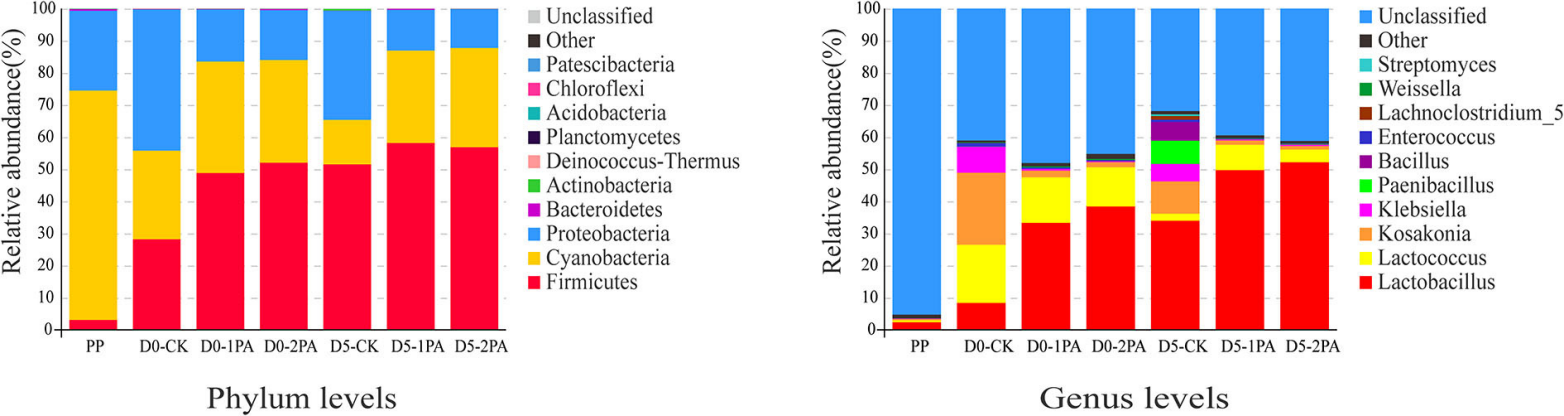
Relative abundance of bacterial communities for Napier grass silages treated without or with 1 and 2% pyroligneous acid after 30 days of ensiling and 5 days of exposure to air. PP, fresh material.

**Figure 5:**
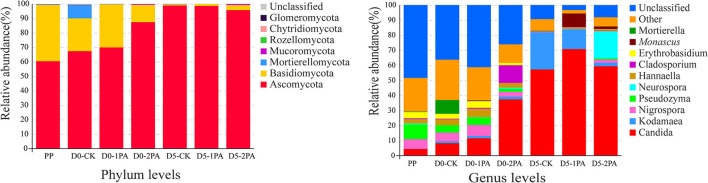
Relative abundance of fungal communities for Napier grass silages treated without or with 1 and 2% pyroligneous acid after 30 days of ensiling and 5 days of exposure to air. PP, fresh material.

The relative abundance of microbial communities after 30 days of ensiling and 5 days of exposure to air is shown in Figures 4, 5, respectively. At the phylum level, the dominant bacteria were *Cyanobacteria, Proteobacteria*, and *Firmicutes* in the fresh Napier grass material, and the relative abundances of were 71.45, 24.80, and 3.04%, respectively. After ensiling, the relative abundance of *Cyanobacteria* decreased, while that of *Firmicutes* increased in all treatment groups. The addition of PA decreased the relative abundance of *Proteobacteria*, when compared with the control. After 30 days of ensiling, the relative abundances of *Lactobacillus, Lactococcus, Kosakonia*, and *Klebsiella* were 8.36, 18.11, 22.40, and 8.10%, respectively, in the untreated silage. The addition of PA increased the relative abundance of *Lactobacillus* and decreased that of *Kosakonia* and *Klebsiella*. After 5 days of exposure to air, PA-treated silage had greater relative abundances of *Lactobacillus* and *Lactococcus* and lower relative abundances of *Lactobacillus, Klebsiella, Paenibacillus*, and *Bacillus* than those in the untreated silage. For fungal communities, *Ascomycota* was the most predominant phylum, followed by *Basidiomycota* before ensiling (Figure 5). After exposure to air, the relative abundance of *Ascomycota* increased, while the relative abundance of *Basidiomycota* decreased in all treatment groups. The addition of PA lowered the abundance of *Mortierellomycota*. On the genus level, the relative abundance of *Candida* increased, while the relative abundance of *Pseudozyma* decreased after 30 days of ensiling, compared with the fresh material. PA application decreased the relative abundance of *Mortierella*. A higher abundance of *Cladosporium* was observed in 2% PA-treated silage than in the control group. After 5 days of exposure to air, the relative abundance of *Kodamaea* decreased, while that of *Monascus* increased with PA addition. Moreover, the high abundance of *Neurospora* was observed in 2% PA-treated silage.

## Discussion

### Characteristics of the fresh Napier grass

In the present study, the DM content was lower than the ideal value (30–35%) of ensiling (Guyader et al., 2018). As Wang et al. (2021) reported, the suitable DM content of the fresh material is necessary to inhibit the fermentation of undesirable microorganisms, mainly *Clostridium*, which would lead to the nutrient loss and the generation of high proportion of NH_3_-N during ensiling. The relatively high number of *Clostridium* might be due to the inadequate DM content. The CP content of Napier grass observed in this study was higher than that determined by Du et al. (2022). The difference might be due to the factors such as climate, location, varieties, harvest time, and plant conditions (Wang et al., 2020). The WSC content and LAB count of the fresh material are two decisive factors to obtain well-preserved silage. In general, 60–70 g/kg of the DM WSC content is required to provide the sufficient fermentation substance (Wang et al., 2019). As dominant bacteria, the LAB count should reach the theoretical requirement (>5.00 log_10_ CFU/g FM) (Wang et al., 2018). But both the WSC content and LAB count did not meet conditions of good fermentation. The numbers of undesirable microorganisms were also relatively high. Therefore, measures should be taken to improve the quality of Napier grass silage.

### Aerobic stability of Napier grass silage

The changing temperature was recognized as a key indicator to estimate the aerobic stability of silage (Drouin et al., 2021). After exposure to air, aerobic fungi grow abundantly and release massive heat in the process of metabolizing and consuming nutrients. In particular, yeasts are regarded as the promoter of aerobic deterioration, the number of which can partly reflect the increased temperature of silage (He et al., 2020b). Therefore, the improvement in aerobic stability of PA-treated silage could be indirectly explained by a reduction in the number of yeasts. With the extension of aerobic exposure time, the temperature of silage could achieve a peak value during the period of the vigorous growth of aerobic fungi (Da Silva et al., 2018). The addition of PA markedly decreased the maximum temperature of silage, which indicates that PA has an important influence on inhibiting the activity and growth of aerobic fungi and improving aerobic stability of silage.

### Fermentation quality of Napier grass silage

The pH value less than 4.2 is the standard for well-preserved silage, which was greatly affected by acid concentration and buffer capacity of the material (Kung et al., 2021). In the present study, the decreased pH value might be due to accumulated lactic acid and acetic acid in PA-treated silage, which effectively inhibited the growth of undesirable microorganisms (coliform bacteria, yeasts, and molds). In the process of fermentation, abundant enzymatic reactions and microbial activities occur (He et al., 2020a). Among these reactions, protein hydrolysis is one of the most important reactions, where TP is converted to non-protein nitrogen (such as small peptides and amino acids free nitrogen) and NH_3_-N (Wang et al., 2021). NH_3_-N, an alkaline substance, is produced by the respiration of plant cells and the metabolism of microorganisms (mainly the metabolism of microorganisms such as coliform bacteria). PA application significantly reduced the NH_3_-N content, indicating that it could effectively decrease proteolysis by direct acidification.

### Microbial diversity of Napier grass silage

The next-generation sequencing technique has been extensively used to detect the composition and abundance of microbial communities in silage (Ni et al., 2017). In the present study, Good's coverage values indicated that most microorganisms were sufficiently captured by sequencing. In addition, the microbial α-diversity of each treatment was evaluated by OTUs (Sobs), richness (Chao1 and Ace indexes), and diversity (Simpson). The addition of PA resulted in the increase in Sobs, Chao1, and Ace values and a decrease in the Simpson value in Napier grass silage, indicating an increase in the richness of bacterial communities in Napier grass silage but a decrease in its diversity. This might show that the higher the abundance of dominant bacteria, the lower the diversity of the bacterial community (Ogunade et al., 2018). Moreover, PA application showed strong anti-fungal property, thus decreasing the richness and diversity of fungal communities of Napier grass, especially in 2% PA treatment. With the increase in the duration of aerobic exposure, the microbial α-diversity of each treatment reduced. Similarly, Zhang et al. (2019b) also found that the α-diversity of fungal communities decreased from day 0 to day 3 of aerobic exposure. However, the change in bacterial α-diversity found int his study was inconsistent with that of our research, and its diversity did not decrease.

The β-diversity of microbial communities was analyzed to compare the difference in the flora structure and species composition among the samples using PCA, a specific analysis tool. A clear separation between PA-treated and untreated silage showed that PA exerted an apparent effect on microbial communities. Moreover, the increase in the PA concentration might promote the variance of the fungal community, whereby resulting in clear segregation between 2% PA-treated and untreated silage. The extended duration of aerobic storage might also influence the β-diversity of fungal communities, which increased the discreteness of all samples.

### Microbial abundance of Napier grass silage

In the process of aerobic storage, the relative abundance of microbial communities changed, which might cause the variation of the chemical composition (Zhang et al., 2021). *Cyanobacteria, Firmicutes*, and *Proteobacteria* were the most prevalent bacterial phyla in silage (Liu et al., 2019). Among these phyla, *Firmicutes* was the prominent bacterium in most grass silage samples, which has a positive effect on hydrolysis and acidogenesis (St-Pierre and Wright, 2014). In the present study, *Cyanobacteria* was the most abundant bacterium detected before ensiling. However, the relative abundance of *Firmicutes* increased after ensiling; especially, the dominant phylum shifted from *Cyanobacteria* to *Firmicutes* in PA-treated silage. Moreover, *Proteobacteria* might have low acid-tolerant ability, and its growth was affected by PA application. Ridwan et al. (2015) reported that *Proteobacteria* could use lactic acid and cause nutrient loss. Thus, PA application might be beneficial to preserve forage nutrition. *Lactobacillus* and *Lactococcus* are commonly used as silage additives, which are can quickly occupy the dominant position after competing with undesirable microorganisms at the early ensiling stage and produce organic acids to ensure good fermentation (Yang et al., 2016). Their high relative abundance might lead to a decreased pH value and improved silage fermentation quality in PA-treated silage. On the contrary, *Bacillus* and *Paenibacillus* are aerobic bacteria, which can rapidly consume organic acids and sugar, and increase the pH value (Graf et al., 2016). The low relative abundances of *Bacillus* and *Paenibacillus* would expectedly improve fermentation quality and aerobic stability in PA-treated silage after 5 days of exposure to air. *Kosakonia*, belonging to *Enterobacteriaceae* family, possesses the characteristics of promoting plant growth, such as nitrogen fixation (Quintas-Nunes et al., 2022), and can also decrease the conversion of molecular nitrogen to NH_3_ and mainly synthesize proteins (Gao et al., 2020). The growth of *Kosakonia* might explain the phenomenon that the untreated silage had a high TP content after 5 days of exposure to air. The content of true protein was higher in the control than in 1% PA- and 2% PA-treated silage. However, *Kosakonia* might have weak acid-resistant ability, and its abundance was reduced in PA-treated Napier grass silage. *Klebsiella* is a Gram-negative facultative anaerobe and is regarded as a harmful bacterium in silage, which can cause inflammation and aerobic spoilage of feed (Lin et al., 2021). The high relative abundance of *Klebsiella* may be one of the reasons for aerobic deterioration in the untreated Napier grass.

Fungi are considered as the main promoters of aerobic deterioration of silage. Understanding the dynamics of fungal composition and their relative abundances is conducive to analyzing the role of different fungal communities and the effects of PA. In the present study, *Ascomycota* and *Basidiomycota* were the most dominant phyla present in the silage samples. Similarly, Romero et al. (2017) found that *Ascomycota* was the predominant fungal phylum before and after ensiling. *Mortierellomycota* is often associated with the increase in the pH value of soil (Shi et al., 2020). It might also explain the higher pH value in the untreated silage after 30 days of ensiling. *Candida* was one of the colonizers in Napier grass, the relative abundance of which was increased after ensiling and aerobic exposure. Khunnamwong et al. (2020) previously reported that three *Candida* species played a major role in the inhibition of *Aspergillus fumigatus* growth due to the fungistatic effect. However, the relevant report also indicated that *Candida* was an undesirable microorganism and could assimilate lactic acid to accelerate the aerobic decay of silage (Liu et al., 2019). But in the present study, the high abundance of *Candida* in PA-treated silage did not accelerate the spoilage of Napier grass silage. Perhaps more research is needed to understand the effect of *Candida* during the aerobic exposure of silage. *Cladosporium* is a prevailing dominant endophytic genus in many plants, which can produce secondary metabolites with antioxidant, antimicrobial, and growth-promoting properties (Chen et al., 2022). Therefore, *Cladosporium* with a high abundance in 2% PA-treated silage might have a positive effect on improving fermentation quality and aerobic stability of silage. *Mortierella* is often detected in over-heated and rotting plant material with pH values of 8–9, which is hardly isolated from good-quality silage or hay (Austwick, 1976). In the present study, *Mortierella* was effectively inhibited by PA after 30 days of ensiling, the relative abundance of which was far below 0.15%. After 5 days of exposure to air, the addition of PA increased the relative abundance of *Monascus*. According to the report of Liu et al. (2022), *Monascus* can produce a variety of nutritional or functional molecules (including small molecular peptides, free amino acids, and ergosterol), which can inhibit other microorganisms. Therefore, *Monascus* might be a beneficial fungus for silage preservation. *Kodamaea*, belonging to the *Ascomycota* phylum, is reported to cause life-threatening infections in humans (Diallo et al., 2019). The high abundance of *Kodamaea* might be undesirable in silage. *Neurospora* can produce xylanase (Liu et al., 2020). The high abundance of *Neurospora* might degrade lignocellulose and provide sufficient fermentation substrate for LAB during ensiling. It might promote LAB fermentation and obtain a lower pH value in 2% PA-treated NG.

## Conclusion

In the present study, PA application decreased the numbers of coliform bacteria, yeasts, and molds; pH value; and NH_3_-N content. PA-treated silage samples were stable during the 5-day aerobic test. The addition of PA increased the relative abundance of *Lactobacillus* and reduced that of *Klebsiella*, and *Kosakonia*. For fungal communities, it also increased the relative abundance of *Candida*. After 5 days of exposure to air, PA application decreased the relative abundance of *Kodamaea* and increased that of *Monascus*. The abundances of *Cladosporium* and *Neurospora* were relatively high in 2% PA-treated NG, while these genera were invisible in the control group. In sum, PA application could improve fermentation characteristics and aerobic stability, as well as alter microbial communities of silage. The addition of 2% PA showed a better effect.

## Data availability statement

The datasets presented in this study can be found in online repositories. The names of the repository/repositories and accession number(s) can be found below: https://submit.ncbi.nlm.nih.gov/subs/bioproject/SUB11801185/ overview; PRJNA827708 https://www.ncbi.nlm.nih.gov/sra/PRJNA827708, PRJNA858920.

## Author contributions

DC and MZ: investigation, software, data curation, formal analysis, and writing—original draft. YZho: investigation, methodology, visualization, and validation. LG and WZ: investigation, methodology, visualization, and validation. WX: revision and validation. MW: conceptualization, data curation, project administration, supervision, and validation. YZhu: conceptualization, funding acquisition, project administration, resources, funding acquisition, and validation. All authors contributed to the article and approved the submitted version.

## Funding

This research was funded by the Guangdong Science Forestry Technology and Innovation Commission (Grant Nos. 2018KJCX001 and 2019KJCX001), National Key R&D Projects (Grant No. 2017YFD0502102-02), and Guangdong Provincial Science and Technology Special Foundation (Grant Nos. 210723106900762 and 2021020103-2).

## Conflict of interest

Author WZ was employed by company Zhengzhi Poultry Industry Co., Ltd. The remaining authors declare that the research was conducted in the absence of any commercial or financial relationships that could be construed as a potential conflict of interest.

## Publisher's note

All claims expressed in this article are solely those of the authors and do not necessarily represent those of their affiliated organizations, or those of the publisher, the editors and the reviewers. Any product that may be evaluated in this article, or claim that may be made by its manufacturer, is not guaranteed or endorsed by the publisher.
